# Analysis of the Welding Type and Filler Metal Influence on Performance of a Regenerated Gear

**DOI:** 10.3390/ma14061496

**Published:** 2021-03-18

**Authors:** Svetislav Marković, Dušan Arsić, Ružica R. Nikolić, Vukić Lazić, Nada Ratković, Branislav Hadzima, Janusz Szmidla, Robert Ulewicz

**Affiliations:** 1 Faculty of Technical Sciences Čačak, University of Kragujevac, 32000 Čačak, Serbia; svetom@mts.rs; 2 Faculty of Engineering, University of Kragujevac, 34000 Kragujevac, Serbia; dusan.arsic@fink.rs (D.A.); vlazic@kg.ac.rs (V.L.); nratkovic@kg.ac.rs (N.R.); 3 Research Center, University of Žilina, 010 26 Žilina, Slovakia; branislav.hadzima@uniza.sk; 4 Department of Mechanics and Machine Design Fundamentals, Czestochowa University of Technology, 42201 Czestochowa, Poland; szmidla@imipkm.pcz.pl; 5 Department of Production Engineering and Safety, Czestochowa University of Technology, 42201 Czestochowa, Poland; robert.ulewicz@pcz.pl

**Keywords:** damages, regenerations, hard facing, spur gears, microstructure, microhardness, teeth flank durability, economic efficiency

## Abstract

This paper presents the results of voluminous experimental investigations conducted to analyze the influence of the welding procedure on the performance of regenerated gears. Cylindrical spur gears were tested, both newly manufactured and regenerated, in two fundamentally different ways: by hard facing (surfacing) with the “hard” filler metal (DUR 600-IG) and with the “soft” filler metal (EVB2CrMo) with subsequent cementation and quenching. The regeneration procedures were defined and executed, while, subsequently, the microstructure and microhardness of the hard-faced layers were established and measured, followed by checking the durability of the hard-faced teeth flanks. Finally, techno-economic analysis was performed to establish the rationality of the conducted regenerations, i.e., the costs of regenerated and newly manufactured teeth were compared. Based on the results of the conducted investigations, it was possible to establish the influence of the welding type on the performance characteristics (primarily the service life) of the regenerated gears. For individual reparatory hard facing, the procedure with the “hard” filler metal exhibited better characteristics, while for batch reparation of numerous damaged gears, the reparation with the “soft” filler metal, followed by cementation and heat treatment, might be more convenient.

## 1. Introduction

The gears transfer the load through the teeth, i.e., the active parts of their flanks. The load transfer is accompanied by the relative movement of the gear and pinion teeth with mutual sliding and rolling. In this way, the stress of the surface layers, caused by the contact (Hertz) pressures, appears on the teeth flanks, while the flanks of the teeth slide at the same time. The bending stress occurs at the teeth base.

Statistical data show that in the gear transmission elements, newly manufactured, most damages appear on gears, followed by failures of bearings, while failures of other parts of the power transmitters are significantly less common. That is the primary reason why many researchers paid special attention to the phenomena of the damage and destruction of gear teeth, their character, the causes for their appearance and the possibility of eliminating those causes [1,2,3,4,5]. A much smaller number of researchers have studied the possibility of repairing those damages, namely, extending the service life of gears and increasing their safety and reliability in operation. By analyzing the causes for loss of the gears’ operational capacity and techno-economic indicators of restoration, one can conclude that it is most expedient to regenerate gears to prolong their service life.

The aim of this paper was to point out the numerous advantages of the regeneration process of damaged gear parts and to show how to carefully select the optimal welding technology and additional material, on a concrete example of reparation by welding. In addition, the authors pointed out the techno-economic benefits coming from application of the regeneration process through numerous parameters, such as exploitational reliability and economic justification. It is pointed out that regeneration can not only restore the function to the part but even achieve better properties than those originally possessed by the base material. This paper’s results can be useful for all the researchers and engineers dealing with these areas to opt for the regeneration of damaged parts more easily, with a detailed analysis of the gear regeneration process. In continuation of the introduction, a brief analysis of works in this field is given.

Durmus et al. investigated the wear resistance of iron-based hard-faced coatings that were produced with Fe-Cr-C-B- and Fe-Cr-C-based filler metal (FM) wires, according to the ball-on-disc and dry sand/rubber wheel wear tests [6]. Iron-based hard facing alloys were coated on St37 steel by arc welding without using a shielding gas. Their results showed that the wear resistance is not only related to the hardness and volume fraction of hard phases but also to the morphology of microstructural constituents. Existence of martensite in the substrate phase raised not only the hardness but also the brittleness, which negatively affects the wear resistance.

Li et al. studied the influence of a rare-earth (RE) oxide addition to the electrode coating for hard facing of a large gear and investigated the microstructures of the hard-faced specimens with and without the RE oxide [7]. During the hard facing process of the damaged gear by the conventional electrode, the large gear had to be heated to about 400 °C before the hard facing and re-heated post-festum, otherwise cracks would appear on the hard-faced metal and in the heat-affected zone (HAZ) of the gear itself. Since for the pre-heating and post-heat treatment of such a large gear (dimension larger than 4 m) a large enough furnace was not available and the hard facing technology was too complicated, they had to develop a new kind of electrode, by which the hard facing of the large gear can be carried out practically without pre-heating or post-heat treatment. They reported that the microstructure of the hard-faced metal was refined due to adding the RE oxide in the electrode coating. No cracks, air holes or other defects in the binding sites between the hard-faced metal and substrate were recorded.

Similar to previous research, Hao et al. developed electrodes for hard facing of medium carbon steel with six additions of a rare-earth (RE) oxide [8]. They studied the microstructure, inclusion and the fractography of the hard-faced metal, by optical and scanning electron microscopy, and analyzed the effects of the rare-earth oxide on the microstructure and inclusions in it. The microstructure of the hard-faced metal was composed of ferrite and a small amount of pearlite and it was refined at first and then coarsened with the increase in the rare-earth oxide addition. The ferrite grain was coarsened because the misfits between Ce_2_O_3_ and d-Fe and Ce_2_O_2_S and d-Fe were increasing with the further increase in the rare-earth oxide addition.

Xing et al. prepared hard facing alloys with different amounts of Ceria by self-shielded flux-cored arc welding [9]. The base material for deposition of the hard facing was the Q235 steel. The flux core powder was composed of a mineral powder and an alloy powder. They carried out the abrasion tests of the hard-faced deposits using the dry sand/rubber wheel machine, measured their hardness and analyzed their microstructure. The results showed that the wear resistance was determined by the size and distribution of the carbides, as well as by the substrate microstructure. The main wear mechanisms, observed at the surfaces, included micro-cutting and micro-ploughing of the substrate. The addition of Ceria improved the hardness and the fracture toughness of the hard-faced deposited layers, which caused an increase in resistance to plastic deformation and scratch, thus improving their wear resistance, as well.

The purpose of the work by Gualco et al. was to study the microstructural evolution and wear resistance of a nanostructured iron-based alloy deposited by the FCAW (flux-cored arc welding) process [10]. They evaluated the effect of the number of layers on the microstructure and wear resistance of a nanostructured iron-based alloy deposited by the FCAW process. The wear resistance was higher for the specimen welded with two layers which could be associated with the increased presence of ultra-hard carbides.

Suraj discussed different methods to evaluate the wear and corrosion resistance properties of mild steels, such as EN-8, EN-9 and EN-24, by calculating their corrosion rate [11]. He used the pin on disc apparatus for analysis of ferrous welded materials, hard-faced by the tungsten inert gas (TIG) welding process, and the Vickers microhardness tester for measuring the microhardness. He found that EN-24 exhibited the least wear when compared to EN-8 and EN-9, since its microhardness was higher than that of the other two materials. The hard-faced materials were more corrosion-resistant than the parent metal. The hardness of the three materials varied in accordance with their chemical composition.

Czuprynski studied the metal-mineral-type abrasive wear of a wear-resistant plate, made by a tubular electrode with a metallic core and an innovative chemical composition, using the manual metal arc hard facing process [12]. The properties of the new layer were compared to the results of eleven wear plates manufactured by global suppliers. Based on the wear resistance tests in laboratory conditions and surface layer hardness tests, the wear plates most suitable for use in the metal-mineral conditions were chosen. The results demonstrated the high metal-mineral abrasive wear resistance of the deposited weld metal produced by the new covered tubular electrode. Results also suggested the high linear relationship between an increase in the surface hardness and an increase in the mineral abrasive resistance of the wear-resistant plate’s hard-faced layers.

Josifović and Marković laid down some theoretical considerations on gears’ regeneration in general, emphasizing advantages of hard facing as the reparation procedure, especially from the aspect of economic effectiveness [13,14].

Lazić et al. conducted an educated selection of hard facing technology, related to the complex procedure of checking the quality of the hard-faced layer [15]. They concluded that the reparatory hard facing operations of various machine parts could be performed only in specialized regeneration workshops. Those should be furnished with adequate equipment and employ the corresponding expert and skilled staff. The estimated financial net benefits of regeneration vs. purchasing the new parts were exceptionally high. They include several items: the regeneration procedure is much cheaper than purchasing the new part; the working life of properly regenerated parts far exceeds the working life of the new parts; the machine downtimes are reduced (the hard facing lasts much shorter than the period of ordering and procuring the new part); assortment and quantity of necessary spare parts are reduced. Similar effects can be expected in other areas such as regeneration of damaged pipes, [16] or other working parts [17].

Marković, on the other hand, also considered the tribological characteristics of gears regenerated by hard facing [18]. His research was focused on the difference in those characteristics when the regeneration hard facing was conducted with the so-called “soft” and “hard” filler metals.

Popovic et al. executed the surface welding of rail steel with self-shielded wire, with different heat inputs, and investigated the influence of the welding heat input on the total impact energy and its components, crack growth rate and fracture mechanisms [19]. They showed that toughness decreased as the heat input increased, but with a temperature decrease, those differences were not so marked. An increase in the heat input led to an increase in the share of trans-granular brittle fracture. They also established that the fatigue life increased with welding heat input increase, while the crack growth resistance decreased in the final deposited layer up to the HAZ, at all heat inputs.

Arsić et al. performed an investigation to show which filler metal is the best for hard facing of parts of the construction mechanization and parts subjected to intensive abrasive wear at stone mines [20]. Since the quality of the surface layer imposes a great influence on the working life of parts, the purpose was to find which filler metal would contribute to extending the working life of parts exposed to intensive wear. The tested hard-faced models were made of the low-carbon steel. Samples were prepared from plates hard-faced with various filler metals and subjected to experimental testing of tribological properties, hardness and microstructure. Research was conducted by using a combination of experimental and theoretical approaches. Results showed that the CrWC 600 alloy was the optimal filler metal for hard facing of the analyzed parts. However, the filler metal E DUR 600 also exhibited good results during the experiments.

Ulewicz and Novy [21] studied the influence of surface fatigue properties of steel S355JR2 in the area of ultra-high cycle numbers (in the range from 6 × 10^10^ to 10 × 10^10^ cycles). Their samples were coated by electrolytic coatings and the effect of those coatings on the fatigue properties of the material under an ultra-high cycle was determined.

Vicen et al. [22] investigated the tribological properties of a nitride layer applied to a low-alloyed steel. The authors reported the results of the experimental work, which included determination of the chemical composition and wear resistance, and Rockwell, Vickers and nano-indentation tests, both of the substrate material—the low-alloyed steel—and the deposited nitride layer. They concluded that applying the nitride layer does not significantly improve the tribological properties of the tested low-alloyed steel samples, and thus they do not recommend it for achieving that purpose.

Trsko et al. [23] tested the influence of the welding process on the creation of tensile residual stresses in the heat-affected zone of high-strength low-alloy (HSLA) steels. Results showed that the welding process caused significant grain coarsening in the heat-affected zone. The microstructural changes were also accompanied by the creation of a tensile residual stress field in the weld metal and heat-affected zone, reaching up to a depth of 4 mm. Tensile residual stresses are well known for acceleration of fatigue crack initiation and, together with coarse grains, can lead to a significant decrease in the fatigue properties of the welded structure.

Vicen, Bronček and Novy [24] presented an investigation dealing with a reduction in the friction coefficient of bearing steel 100Cr6. The reduction in friction was realized by the CarbonX DLC (Diamond Like Carbon) coating, which exhibited excellent friction and mechanical properties. Reducing the friction of 100Cr6 bearing steel resulted in reduced wear and an increased lifetime.

## 2. Technological Procedure of Gear Regeneration

Basically, the technological procedure of a gear’s regeneration consists of the following operations [25]:

- Cleaning, washing and degreasing of the gear;
- Identification of the type, size, character and position of the damage;
- Classification of the gear, based on comparison of the damage detection results and requirements from the technical documentation to correct gears intended for regeneration and for rejection (scrap);
- Selection of the optimal regeneration method;
- Defining the regeneration technological procedure;
- Preparation for regeneration;
- Executing the prescribed regeneration procedure;
- Machining the regenerated surfaces;
- Performing the strengthening procedures of the teeth working parts and other functional surfaces;
- Final machining—grinding;
- Final control and testing of the regenerated gear.

## 3. Preparation of Samples for Experimental Testing

Previous research in the field of gear regeneration has shown that reparatory hard facing of the damaged working surfaces is the most reliable method for the renewal of the shapes, dimensions and working performances of the teeth. For the proper selection of an adequate method for reparatory hard facing, it is necessary to know the mechanisms of the contact surfaces’ wear. The regeneration itself is a complex process consisting of numerous technological operations, whose order of execution is particularly important and precisely defined.

The cylindrical spur gears, of the following characteristics, were used as samples for testing [26]:

- Material for manufacturing—steel 20MnCr5;
- Module m = 6 mm;
- Number of teeth z = 43;
- Base profile angle α = 20°;
- Tooth profile angle β = 0°;
- Profile correction x_m_ = 0;
- Root circle diameter d_f_ = 243.6 mm;
- Circular pitch 18.84 mm.

The base material, used for samples in these experiments, was steel for cementation 20MnCr5, whose marks according to different standards and chemical composition are given in Table 1, [26].

Two essentially different procedures of the reparatory hard facing were used for the gear regeneration:

- Hard facing by the “hard” filler metal DUR 600-IG (according to EN14700-EFe8) [27];
- Hard facing by the “soft” filler metal EVB2CrMo (according to EN1599-ECrMo2 B 42) with subsequent cementation and heat treatment, [28].

The gear regeneration through hard facing by the TIG procedure, with DUR 600-IG, consists of the following processes:

- Removal of the cemented layer from the active working surfaces by grinding to the depths of 1.2 + 0.2 and 12 mm along the tooth height;
- Preheating of the gears to a temperature of 230 °C and holding at that temperature for 2 h;
- The TIG hard facing of the prepared surfaces in the argon-protected atmosphere with the DUR 600-IG wire of diameter Ø1.2 mm;
- Returning of the hard-faced gear into the furnace and slow cooling with it;
- Grinding of the hard-faced teeth on the gear-grinding machine “Niles”.

In the case of regeneration by the “soft” filler metal EVB2CrMo, the technological procedure of regeneration is significantly different from the previously described one. It consists of the following operations:

- Soft annealing—this operation is necessary to reduce the surface hardness of the teeth working surfaces and to enable preparation by milling. Annealing was conducted in vacuum, heating up to a temperature of 680 °C, at a rate of 10 °C/min. The gears were then held at that temperature for 4 h and then cooled at a rate of 2 °C/min.
- Removal of the cemented layer from the teeth active working surfaces by milling on the universal milling machine, to the depths of 1.2 + 0.2 and 12 mm along the tooth height.
- Preheating of the gears to a temperature of 230 °C and holding at that temperature for 2 h.
- Drying of electrodes at a temperature of 350 °C for 4 h.
- The Manual Metal Arc (MMA) hard facing of the prepared surfaces by the cored electrodes, EVB2CrMo.
- High annealing in the furnace.
- Milling to the over-dimension (with addition for grinding) on the gear milling machine Pfauter.
- Soft annealing.
- Cementation in the CO_2_ + CO mixture to the depth 1.0 + 0.1 mm with cooling in the pit.
- Grinding of the hard-faced teeth to nominal size.

During the hard facing, the thickness of the hard-faced layer had to be increased for the size of addition predicted for subsequent surface machining.

## 4. Investigation of Microstructure

**Figure 1:**
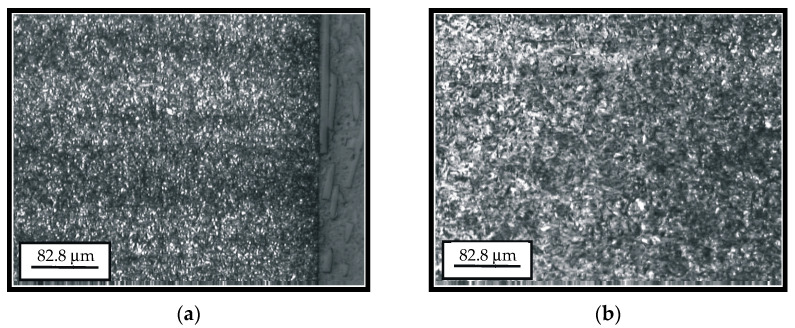
Microstructure of the cemented layer (**a**) and the transient zone (**b**).

The metallographic investigations were conducted on the quantitative optical metallographic microscope POLYVAR-MET (Leica Reichart-Jung, Wien, Austria) with magnifications of 20 to 2000 times [26]. Figure 1 presents the microstructure of the cemented layer and of the transient zone of the hard-faced tooth.

The microstructure of the surface and subsurface layers (cemented layer and the core of the newly manufactured tooth) is presented in Figure 1a. The basic micro-constituent of the tooth cemented layer is the tempered martensite (dark areas in the figure). The smaller share of the retained austenite can also be noticed. In the surface zone, up to the depth of 0.2 to 0.3 mm, individual coarser carbides of the alloying elements are also present. Based on the appearance of the martensite needles, one can conclude that the material possesses a fine-grained structure, which points to the fact that the heat treatment process was properly conducted. Based on Figure 1b, one can see the difference between the microstructures of the cemented layer (the right-hand portion of the figure), microstructure of the transient zone (the middle portion of the figure) and microstructure of the core (the left-hand portion of the figure). Individual ferrite grains can be noticed in the transient zone (the coarser brighter areas), the number of which is much bigger in the core.

**Figure 2:**
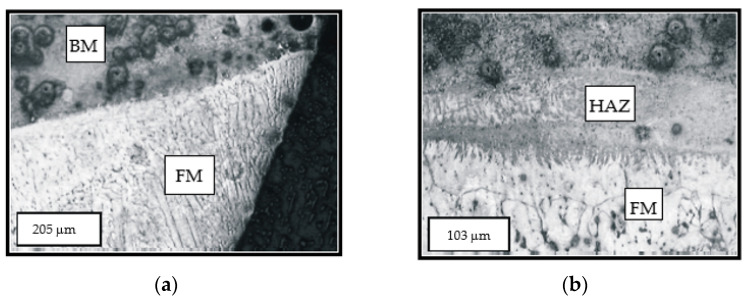
The two-layer hard-faced tooth top—the base metal (BM) and the filler metal (FM) (**a**); the transient line between the filler metal (FM) and heat-affected zone (HAZ) (**b**).

The microstructure of the tooth hard-faced by the TIG procedure and the filler metal DUR 600-IG in the argon protective atmosphere is shown in Figure 2a, while in Figure 2b, one can see the transition from the surfaced layer and the heat-affected zone. In the first figure, one can clearly notice the cast dendritic (stick-like) structure of the welded metal. The more prominent dendritic branches are present in the second surfaced layer, unlike the first layer where, due to the heat input during the second layer deposition, a smaller homogenization of the microstructure occurred. The dendrites’ growth direction, with respect to the base metal (BM) and the first deposited layer, is perpendicular to the tooth surface.

**Figure 3:**
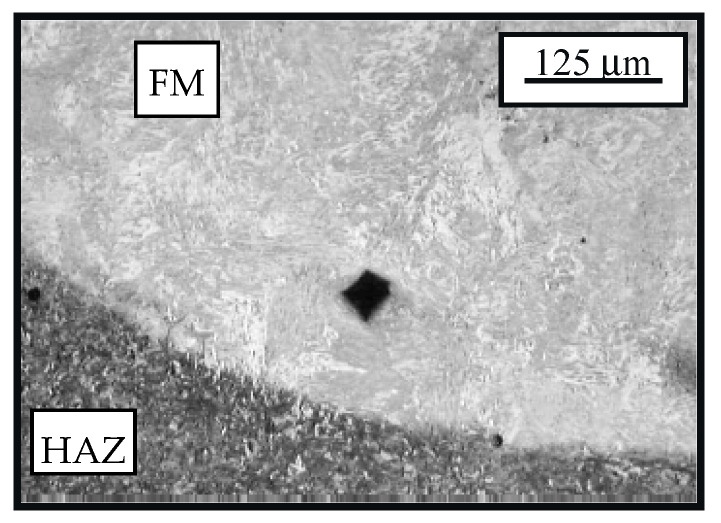
Microstructure of the transient zone from the cemented (bright field) hard-faced layer with the filler metal EVB2CrMo (FM) and the heat affected zone (HAZ) of the base metal (BM) (dark field)—state: hard-faced + soft annealed + cemented + heat treated.

The microstructure of the cemented layer hard-faced with the filler metal EVB2CrMo is shown in Figure 3. The basic micro-constituent in the hard-faced layer zone is the tempered martensite. Based on the degree of etched material, one can conclude that there is a significant difference in the chemical composition between the FM and BM.

**Figure 4:**
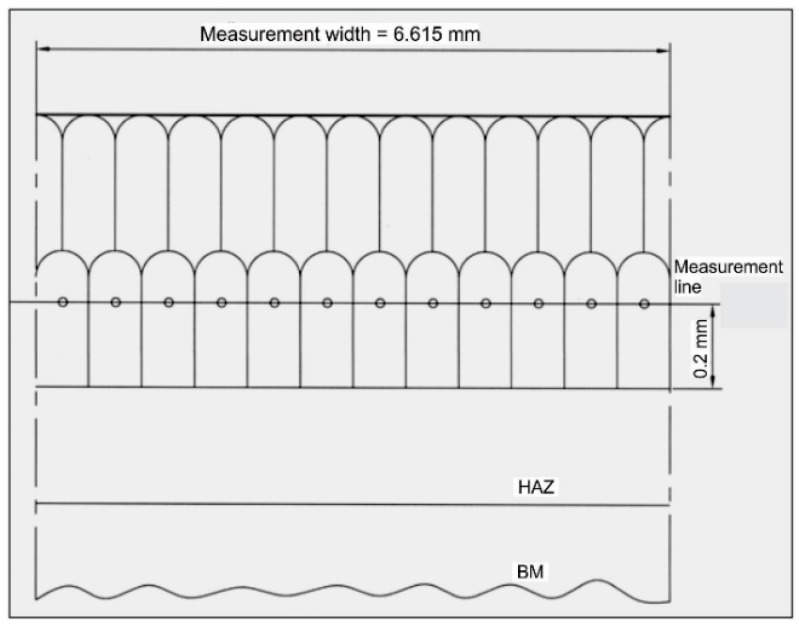
The measurement line of the dendritic crystals’ medium width.

**Figure 5:**
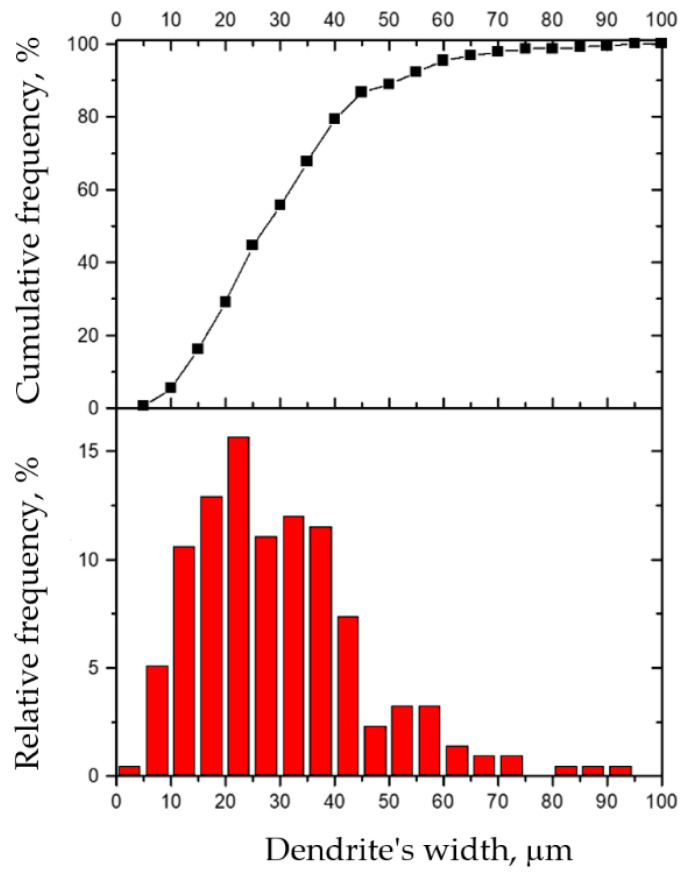
Cumulative and relative frequencies of dendrites’ widths in the hard-faced layer deposited by the DUR 600-IG filler metal.

Since the exploitation characteristics of the hard-faced layer depend, to a great extent, on the size of dendrites within it, the dendritic crystals’ width was measured on the optical microscope [26]. Those measurements were performed at a distance of 0.2 mm from the joint line, as shown in Figure 4. A total of 217 dendritic crystals were measured over the measurement width of 6.615 mm. The measurement results are presented in Table 2 and in Figure 5 [29].

Based on the results presented in Table 2 and the diagrams in Figure 5, one can see that the average width of the dendritic crystals is 30.48 μm. From the histograms, one can also notice that about 30% of dendrites have an average width of up to 20 μm. About 45% of dendrites have an average width of up to 30 μm, while about 80% have an average width of up to 40 μm. A width of over 60 μm is only achieved in about 3% of dendrites. The largest number of dendrites, i.e., 15.66% of them, have a width of 20 to 25 μm, while 73.7% of dendrites have a width within the range of 10 to 40 μm. Considering these results, one can conclude that the liquid bath during the hard facing had a relatively small volume and that the hard-faced layer possesses a fine-grained dendritic structure [26].

## 5. Investigation of Microhardness

**Figure 6:**
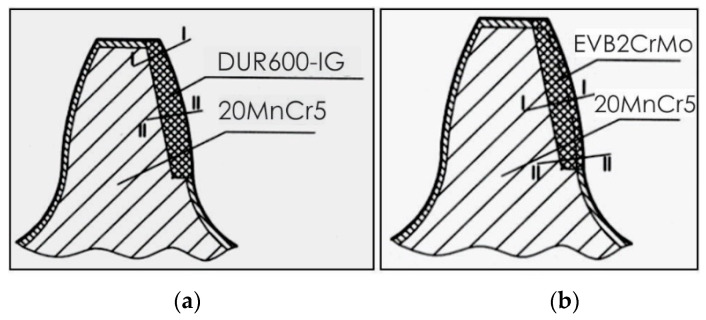
Microhardness measurements’ directions: tooth hard-faced with the DUR 600-IG FM (**a**) and tooth hard-faced with the EVB2CrMo FM (**b**).

**Figure 7:**
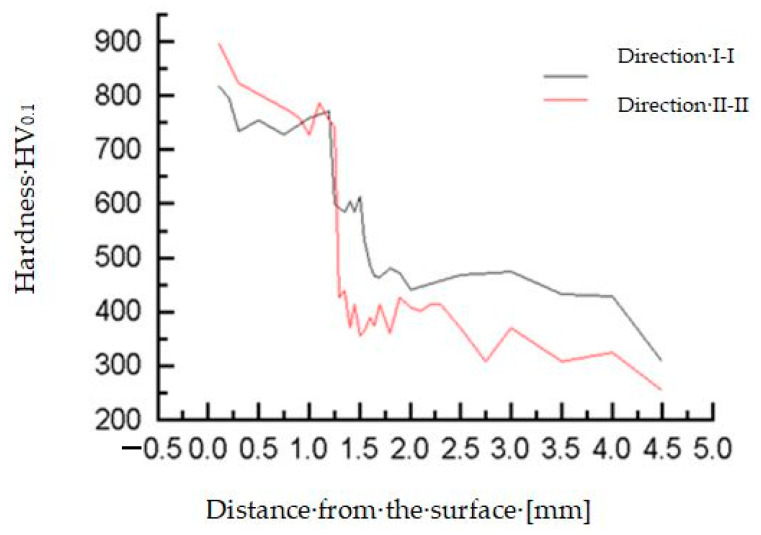
Microhardness measurement results for the tooth hard-faced with FM DUR 600-IG.

**Figure 8:**
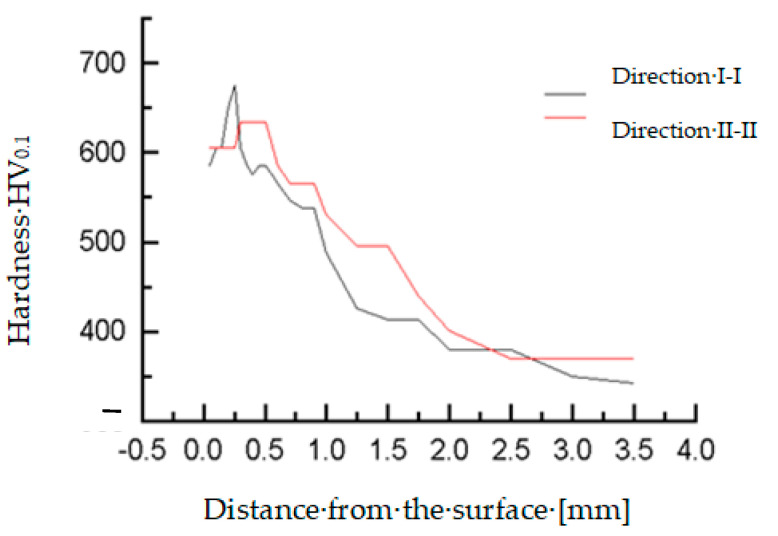
Microhardness measurement results for the tooth hard-faced with FM EVB2CrMo.

The microhardness of the teeth, hard-faced by the TIG procedure with the filler metal DUR 600-IG, was measured in two directions, close to the top of the tooth and in the zone of the pitch circle, Figure 6a. Results are presented in Table 3 and Table 4, as well as in diagrams in Figure 7 [18]. For the tooth hard-faced with the filler metal EVB2CrMo, hardness was also measured in two directions, but in the zones of the pitch circle and the lowest area of the hard-faced layer, Figure 6b. Measurement results are presented in Table 5 and Table 6, as well as in Figure 8.

**Figure 9:**
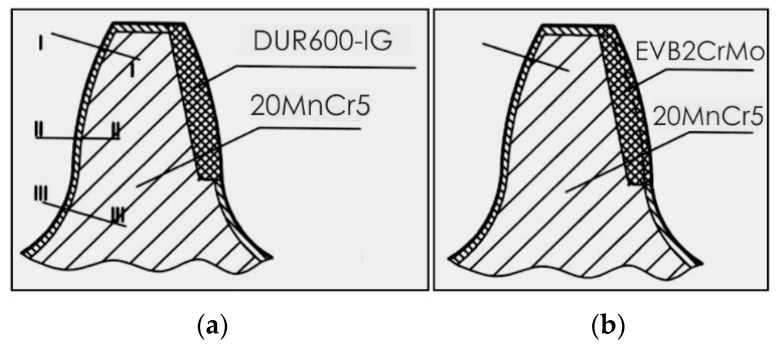
Microhardness measurements’ directions on the inactive tooth flanks: tooth hard-faced with the DUR 600-IG FM (**a**) and tooth hard-faced with the EVB2CrMo FM (**b**).

**Figure 10:**
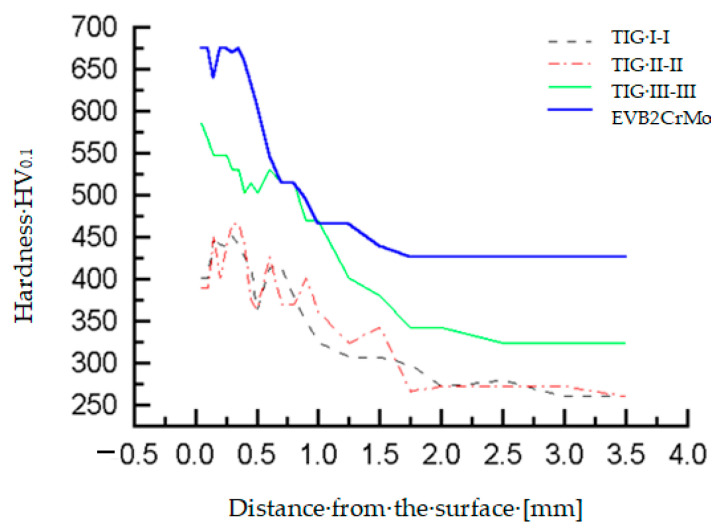
Microhardness measurement results on the inactive tooth flanks for the teeth hard-faced with FM DUR 600-IG and EVB2CrMo.

Besides the conducted investigations, the microhardness of the inactive tooth flanks of the regenerated teeth was measured, as well. For the sample hard-faced with the DUR 600-IG filler metal, hardness was measured in three directions, close to the tooth top (I-I), in the pitch circle zone (II-II) and at the tooth base (III-III), Figure 9a, while for the tooth hard-faced with the EVB2CrMo filler metal, hardness was measured only in the middle of the tooth, Figure 9b. Measurements’ results are presented in Table 7, Table 8, Table 9 and Table 10, as well as in Figure 10.

From the diagram in Figure 10, it is obvious that a hardness reduction occurs on the non-hard-faced surfaces of the teeth. From the presented results, one can easily notice that the largest hardness reduction occurs for the tooth hard-faced by the TIG procedure with the DUR 600-IG filler metal at the tooth head, while the tooth base (where there is much more material and which is further from the heat source during the hard facing) had the lowest hardness reduction. Such results were expected and are quite logical [26].

## 6. Investigation of the Tooth Flanks’ Durability

**Figure 11:**
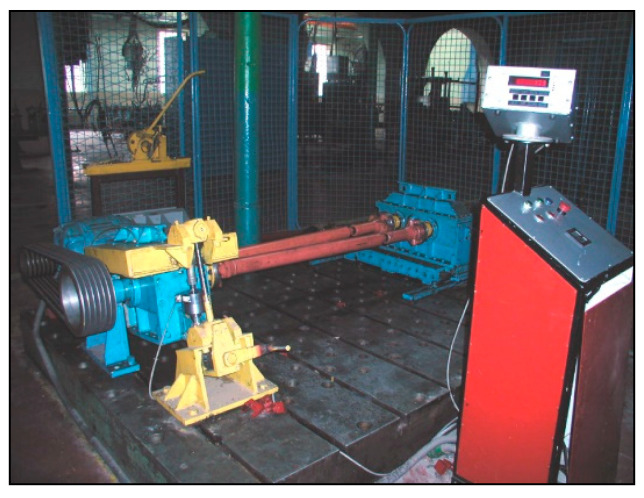
Gears’ testing device with closed circuit and reactive loader.

Investigation of the regenerated and newly manufactured teeth flanks’ durability was performed on a closed-circuit device shown in Figure 11 [27]. The workload of 3000 Nm was introduced to gears gradually. In that way, failures were avoided.

**Figure 12:**
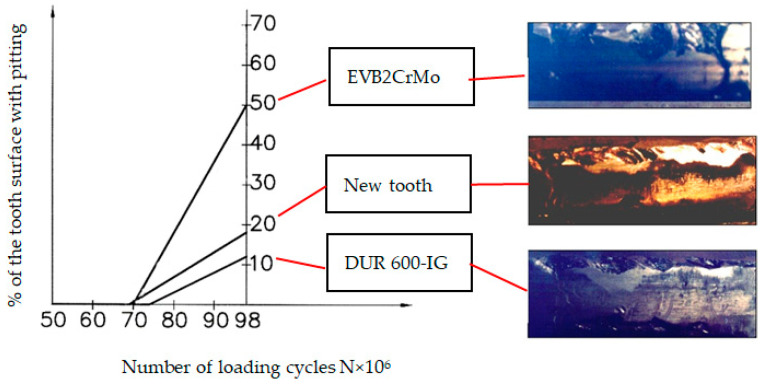
Destructive pitting development rate.

The appearance of the destructive wear, namely, the first pitting pits, was noticed after 69 × 10^6^ loading cycles for the newly manufactured teeth and for the teeth hard-faced with the EVB2CrMo filler metal after 70 × 10^6^ cycles. The longest operation prior to the appearance of the first pits was exhibited by teeth hard-faced by the TIG procedure and the DUR 600-IG filler metal, where the destructive pitting started after 74 × 10^6^ loading cycles. After 98 × 10^6^ loading cycles, the full duration of tests, for newly manufactured teeth, 18% of the most endangered tooth surface was covered with pitting; for the tooth hard-faced with the EVB2CrMo filler metal, the attacked area was about 50 %; and for the tooth hard-faced by the TIG procedure, this was only about 12 %. Analyzing the operation time until the first appearance of the destructive pitting of the regenerated and newly manufactured teeth produced the diagram shown in Figure 12.

The size of the pitted surface of the tooth was determined by the visual method. The template was made of opaque foil, the opening of which was as long as the tooth’s height (about 17.5 [mm]) and 10 [mm] wide. Then, the teeth portions were examined with the magnifying glass (magnification 5×). The assessment was made according to the most worn teeth and their parts with the most pits. Those tests were performed in the phase of destructive pitting, namely, after noticing the initial pits with the naked eye.

## 7. Techno-Economic Analysis of Applied Regeneration Procedures

Economic analysis of the applied regeneration procedures assumes a comparison of costs of the regenerated and newly manufactured gears. The regeneration price includes material and labor costs, as well as overheads. The comparison results are presented in Table 11. Analysis is performed through the following parameters [27]:

Indicator of economic justification of regeneration (*k_e_*)—represents the relative price of the regenerated gear (*C_r_*) with respect to price of the new gear (*C_n_*) and is determined according to expression:

(1) $$k_{e}=\frac{C_{n}-C_{r}}{C_{n}}\cdot 100(\%).$$

Assuming that 5% of the teeth working surfaces being affected by pitting are a criterion for interruption of the gears’ working capacity (i.e., the wear limit), the operating time of the gears regenerated by the appropriate welding method (*t_r_*) was determined from the diagram shown in Figure 12. The operation time (service life) of the new gear, until reaching the limit wear, is *t_n_* = 1510 h. 

Coefficient of the exploitational reliability (*k_ep_*)—represents the ratio of the regenerated and new gear service lives:

(2) $$k_{ep}=\frac{t_{r}}{n_{n}}.$$

Condition for economic rationality of regeneration (*β*)—takes into account both the regeneration price and the service life of regenerated teeth until the limit wear appearance. It is calculated according to

(3) $$\beta =\frac{C_{n}\cdot t_{r}}{C_{r}\cdot t_{n}}.$$

Regeneration is justified when the coefficient β is greater than 1. Individual values of all the factors mentioned above, for both regeneration procedures are given in Table 11, [26].

## 8. Conclusions

Improvement of all the mechanical properties of regenerated gears can be achieved through the careful and well-organized preparation of hard facing, selection of the adequate filler metals and the welding method, strict obedience to the prescribed working regimes and by precisely defined and carefully executed heat treatment processes of the regenerated gears’ working surfaces. Standard equipment, available in any metal working shop, can be used for regeneration, which makes the investment costs minimal.

From the aspect of the teeth flanks’ durability, namely, resistance to fatigue wear, the best characteristics possess teeth regenerated by the TIG procedure with the DUR 600-IG filler metal. Somewhat worse properties were obtained for teeth regenerated by the EVB2CrMo filler metal, which were subsequently cemented and heat treated. However, their service life was also longer than expected. Considering the calculated coefficients of economic justification/rationality of the regeneration procedures, one can conclude that the best reparatory hard facing is by the TIG procedure and DUR 600-IG filler metal.

In individual regeneration of gears, somewhat more convenient is hard facing with “hard” filler metals. However, if batch regeneration of the damaged gears would be required (as well as other machine parts), it would be more convenient to apply the “soft” filler metals with subsequent cementation and heat treatment.

## Figures and Tables

**Table 1:** Chemical composition of the base metal.

Steel Mark (Designation)						
ISO DIS	JUS (SRPS)	DIN		GOST	US	
		17,007	17,006		AISI/SAE	UNS
≈20 MnCr5	Č4321	1.7147	20 MnCr5	18 HGT	4820	G48200
Alloying Elements (%)						
C	Si	Mn	P_max_	S_max_	Cr	
0.17–0.22	0.15–0.40	1.1–1.4	0.035	0.035	1.0–1.3	

**Table 2:** Width of the dendritic crystals in the hard-faced layer deposited by the DUR 600-IG filler metal.

Class	Width μm	Count	Cumulative Frequency, %	Relative Frequency, %
0	5	1	0.46083	0.46083
5	10	11	5.52995	5.06912
10	15	23	16.12903	10.59908
15	20	28	29.03226	12.90323
20	25	34	44.70046	15.66820
25	30	24	55.76037	11.05991
30	35	26	67.74194	11.98157
35	40	25	79.26267	11.52074
40	45	16	86.63594	7.37327
45	50	5	88.94009	2.30415
50	55	7	92.16590	3.22581
55	60	7	95.39171	3.22581
60	65	3	96.77419	1.38249
65	70	2	97.69585	0.92166
70	75	2	98.61751	0.92166
75	80	0	98.61751	0.00000
80	85	1	99.07834	0.46083
85	90	1	99.53917	0.46083
90	95	1	100	0.46083
95	100	0	100	0.00000

**Table 3:** Microhardness of tooth hard-faced with the DUR 600-IG FM—direction I-I.

	Hard-Faced Layer							Joint	Heat-Affected Zone (HAZ)				
Distance from the surface (mm)	0.1	0.2	0.3	0.5	0.75	1.0	1.2	1.25	1.3	1.35	1.4	1.45	1.5
Hardness HV_0.1_	818	797	734	755	728	758	772	599	593	585	606	585	613
Corresponds to HRC	64.6	63.9	61.5	62.3	61.2	62.4	63.0	55.1	54.8	54.4	55.5	54.4	55.8
	**Heat-Affected Zone (HAZ)**												
Distance from the surface (mm)	1.55	1.6	1.65	1.7	1.8	1.9	2.0	2.5	3.0	3.5	4.0	4.5	
Hardness HV_0.1_	533	486	465	463	480	472	442	468	475	432	429	309	
Corresponds to HRC	51.2	48.1	46.5	46.3	47.7	47.1	44.7	46.8	47.3	43.8	43.5	30.9	

**Table 4:** Microhardness of tooth hard-faced with the DUR 600-IG FM—direction II-II.

	Hard-Faced Layer							Joint	Heat-Affected Zone (HAZ)						
Distance from the surface (mm)	0.1	0.3	0.8	0.9	1.0	1.1	1.2	1.25	1.3	1.35	1.4	1.45	1.5	1.55	
Hardness HV_0.1_	895	824	772	758	728	786	755	743	426	439	370	414	355	364	
Corresponds to HRC	66.8	64.8	63.0	62.4	61.2	63.5	62.3	61.9	43.2	44.4	37.7	42.2	36.0	37.0	
	**Heat-Affected Zone (HAZ)**														
Distance from the surface (mm)	1.6	1.65	1.7	1.8	1.9	2.0	2.1	2.2	2.3	2.5	2.75	3.0	3.5	4.0	4.5
Hardness HV_0.1_	390	375	413	360	426	408	402	414	415	370	307	370	307	324	254
Corresponds to HRC	39.8	38.1	42.1	36.6	43.2	41.6	41.0	42.2	42.3	37.7	30.7	37.7	30.7	32.6	22.9

**Table 5:** Microhardness of tooth hard-faced with EVB2CrMo—direction I-I.

Distance from the Surface (mm)	0.05	0.1	0.15	0.2	0.25	0.3	0.35	0.4	0.45	0.5	0.6
Hardness HV_0.1_	585	606	606	650	675	606	585	575	585	585	566
Corresponds to HRC	54.4	55.5	55.5	57.8	59.0	55.5	54.4	53.8	54.4	54.4	53.3
Distance from the surface (mm)	0.7	0.8	0.9	1.0	1.25	1.5	1.75	2.0	2.5	3.0	3.5
Hardness HV_0.1_	547	538	538	488	426	414	414	380	380	350	342
Corresponds to HRC	52.1	51.6	51.6	48.2	43.2	42.2	42.2	38.8	38.8	35.5	34.6

**Table 6:** Microhardness of tooth hard-faced with EVB2CrMo—direction II-II.

Distance from the Surface (mm)	0.05	0.1	0.15	0.2	0.25	0.3	0.35	0.4	0.45	0.5	0.6
Hardness HV_0.1_	606	606	606	606	606	634	634	634	634	634	585
Corresponds to HRC	55.5	55.5	55.5	55.5	55.5	57.0	57.0	57.0	57.0	57.0	54.4
Distance from the surface (mm)	0.7	0.8	0.9	1.0	1.25	1.5	1.75	2.0	2.5	3.0	3.5
Hardness HV_0.1_	566	566	566	530	496	496	440	401	370	370	370
Corresponds to HRC	53.3	53.3	53.3	51.1	48.8	48.8	44.5	40.9	37.7	37.7	37.7

**Table 7:** Microhardness of the inactive tooth flank of the tooth hard-faced with the DUR 600-IG FM—direction I-I.

Distance from the Surface (mm)	0.05	0.1	0.15	0.2	0.25	0.3	0.35	0.4	0.45	0.5	0.6
Hardness HV_0.1_	401	401	452	440	440	452	440	426	414	364	414
Corresponds to HRC	40.9	40.9	45.5	44.5	44.5	45.5	44.5	43.2	42.2	37.0	42.2
Distance from the surface (mm)	0.7	0.8	0.9	1.0	1.25	1.5	1.75	2.0	2.5	3.0	3.5
Hardness HV_0.1_	414	380	350	324	307	307	297	272	279	260	260
Corresponds to HRC	42.2	38.8	35.5	32.6	30.7	30.7	29.5	25.9	27.0	24.0	24.0

**Table 8:** Microhardness of the inactive tooth flank of the tooth hard-faced with the DUR 600-IG FM—direction I-II.

Distance from the Surface (mm)	0.05	0.1	0.15	0.2	0.25	0.3	0.35	0.4	0.45	0.5	0.6
Hardness HV_0.1_	390	390	452	401	440	466	466	440	375	364	426
Corresponds to HRC	39.8	39.8	45.5	40.9	44.5	46.5	46.5	44.5	38.1	37.0	43.2
Distance from the surface (mm)	0.7	0.8	0.9	1.0	1.25	1.5	1.75	2.0	2.5	3.0	3.5
Hardness HV_0.1_	370	370	401	360	324	342	266	272	272	272	260
Corresponds to HRC	37.7	37.7	40.9	36.6	32.6	34.6	25.1	25.9	25.9	25.9	24.0

**Table 9:** Microhardness of the inactive tooth flank of the tooth hard-faced with the DUR 600-IG FM—direction III-III.

Distance from the Surface (mm)	0.05	0.1	0.15	0.2	0.25	0.3	0.35	0.4	0.45	0.5	0.6
Hardness HV_0.1_	585	566	547	547	547	530	530	502	514	502	530
Corresponds to HRC	54.4	53.3	52.1	52.1	52.1	51.1	51.1	49.2	50.1	49.2	51.1
Distance from the surface (mm)	0.7	0.8	0.9	1.0	1.25	1.5	1.75	2.0	2.5	3.0	3.5
Hardness HV_0.1_	514	514	470	470	401	380	342	342	324	324	324
Corresponds to HRC	50.1	50.1	46.9	46.9	40.9	38.8	34.6	34.6	32.6	32.6	32.6

**Table 10:** Microhardness of the inactive tooth flank of the tooth hard-faced with the EVB2CrMo FM.

Distance from the Surface (mm)	0.05	0.1	0.15	0.2	0.25	0.3	0.35	0.4	0.45	0.5	0.6
Hardness HV_0.1_	675	675	640	675	675	670	675	660	634	606	547
Corresponds to HRC	59.0	59.0	57.3	59.0	59.0	58.8	59.0	58.3	57.0	55.5	52.2
Distance from the surface (mm)	0.7	0.8	0.9	1.0	1.25	1.5	1.75	2.0	2.5	3.0	3.5
Hardness HV_0.1_	514	514	496	466	466	440	426	426	426	426	426
Corresponds to HRC	50.1	50.1	48.8	46.5	46.5	44.5	43.2	43.2	43.2	43.2	43.2

**Table 11:** Parameters of the techno-economic justification of the applied regeneration procedures.

Regeneration Procedure	*k_e_* (%)	*t_r_* (h)	*k_ep_*	*β*
TIG hard facing with FM DUR 600-IG	34.0	1650	1.09	1.655
MMA hard facing with FM EVB2CrMo + C + HT	36.9	1430	0.95	1.500

## Data Availability

The data presented in this study are available on request from the corresponding author. The data are not publicly available due to possibility for use in further research.
